# Supplementary figures and images for: Robust 3D Pose estimation and Parkinson’s Disease classification via Dual-Stage Adaptive Temporal Perception and graph topology modeling network
Source: PLoS One. 2026 Mar 19;21(3):e0344375. doi: 10.1371/journal.pone.0344375 (PMC13001963; doi:10.1371/journal.pone.0344375)

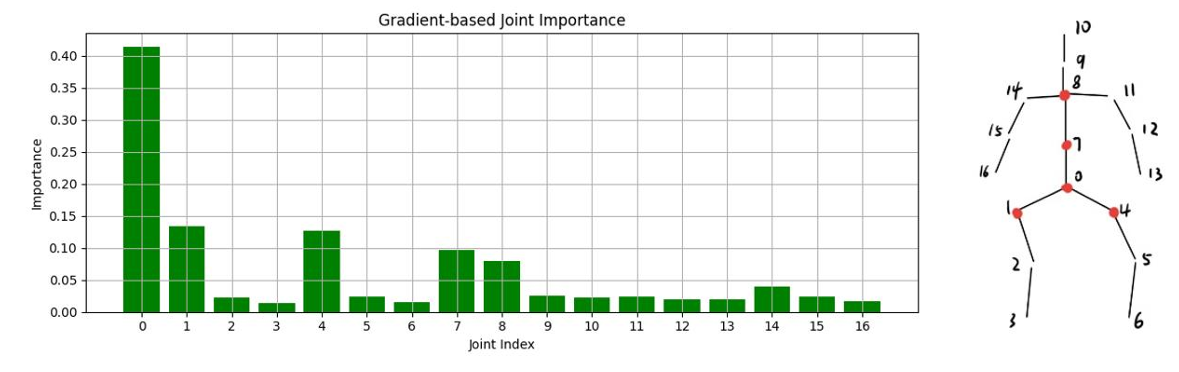

Supplement: S1 Fig — As shown in the Fig, the skeletal key-points that have a large impact on the decision-making results of the model are mainly concentrated in nodes 0, 1, 4, 7, and 8, which correspond to the parts of the human body that are the spine, the thorax, and the hips, respectively. (TIF) [file pone.0344375.s001.tif]

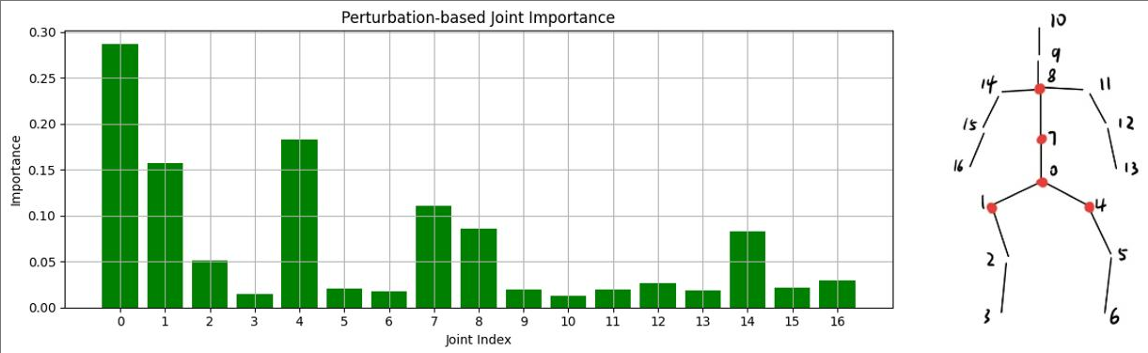

Supplement: S2 Fig — Fig demonstrates the results of the perturbation-based interpretability analysis on the datasets with a score of 1. Through this analysis, it is also found that the key nodes such as spine, chest and hips have a strong influence on the final prediction results These nodes highly overlap with the joint locations in the gradient-based analysis results, indicating that they play a decisive role in making a judgment on the severity of the disease on the datasets with score 1. (TIF) [file pone.0344375.s002.tif]

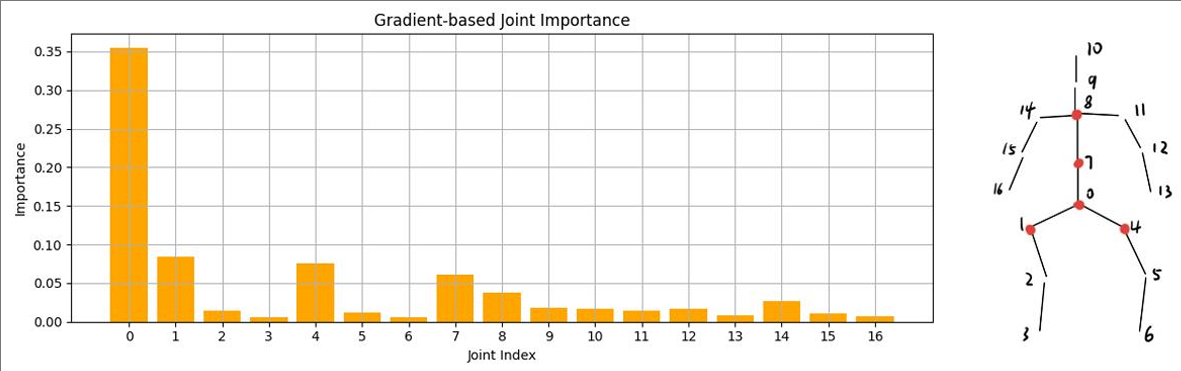

Supplement: S3 Fig — Fig shows the results of the gradient-based interpretability analysis on the datasets with a score of 2. As shown in the fig, the key points of the skeleton that have a large influence on the model’s decision-making results are mainly concentrated in nodes 0, 1, 4, 7, and 8, which correspond to the parts of the human body that are the spine, the thorax, and the hips, respectively. (TIF) [file pone.0344375.s003.tif]

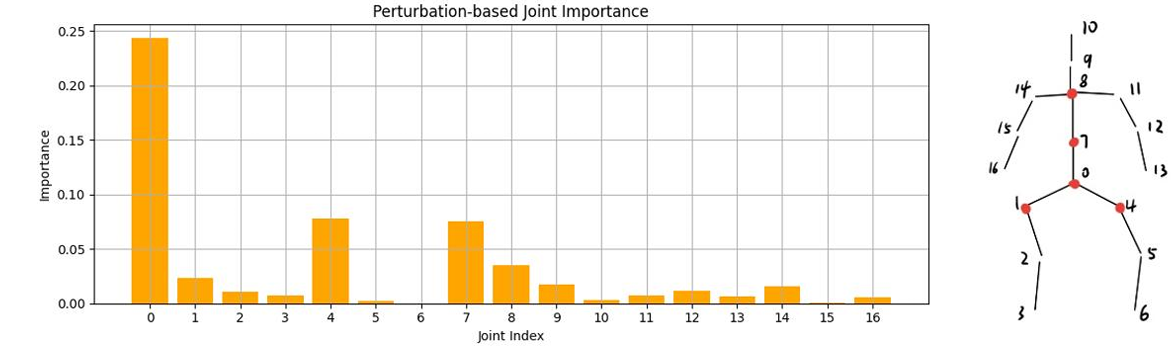

Supplement: S4 Fig — Fig demonstrates the results of the perturbation-based interpretability analysis on the datasets with a score of 2. This analysis reveals that the key nodes, such as the spine, chest, and hips, have a greater impact on the final prediction results. These nodes highly overlapped with the joint locations in the gradient-based analysis results, indicating that they played a decisive role in the judgment of disease severity. (TIF) [file pone.0344375.s004.tif]
