# Supplementary material for: Robust 3D Pose estimation and Parkinson’s Disease classification via Dual-Stage Adaptive Temporal Perception and graph topology modeling network
Source: PLoS One. 2026 Mar 19;21(3):e0344375. doi: 10.1371/journal.pone.0344375 (PMC13001963; doi:10.1371/journal.pone.0344375)
Supplement: Attachment — Submitted filename: Response to Reviewers.docx [file pone.0344375.s005.docx]

**For research article**

Response to Reviewer 1 Comments

**Point-by-point response to Comments and Suggestions for Authors**

**Comments 1:**Introduction requires restructuring for clinical accessibility

**Response 1**: We have completely rewritten the Introduction.

We removed the discussion on MCI and non-motor symptoms. The new introduction now starts by highlighting the limitations of subjective clinical scales (MDS-UPDRS) and the specific challenges of using AI in clinical settings (e.g., occlusion). We also reframed the background to focus on motor subtypes, specifically referencing the importance of quantifying axial motor impairment and postural instability, which aligns with our chosen task (Item 3.9).

**Comments 2:** Benchmarks used for comparison require clearer explanation

**Response 2:** We acknowledge that the baseline methods were not adequately introduced to clinical readers.

We have added a new paragraph at the beginning of the Results section. In this paragraph, we explicitly explain that we selected representative CNN-based (e.g., T3D-CNN) and Transformer-based (e.g., MHFormer) methods to benchmark our model. We clarify that these were chosen to demonstrate our model's specific advantage in handling "missing joints" and occlusion, which are common in real-world clinical videos.

**Comments 3:** Clinical description of the REMAP dataset should be expanded

**Response 3:** We agree that the REMAP dataset description lacked sufficient clinical context.We have significantly expanded the "REMAP Open Datasets" section. We added details regarding:

Sensor Setup: Specified the use of markerless Microsoft Kinect sensors (non-invasive).

Data Volume: Clarified the use of 403 Sit-to-Stand episodes.

Clinical Annotation: Detailed the MDS-UPDRS Item 3.9 scoring criteria (0-4 scale).

Medication Status: Explicitly noted that the dataset records patients in both "On" and "Off" medication states, which is crucial for symptom analysis.

**Comments 4:** TRI-POSE-Net and HumanEva-I appear abruptly in the Discussion

**Response 4:** We sincerely apologize for this oversight. This text was included due to a clerical error and is unrelated to the current study.

We have deleted the entire section regarding "TRI-POSE-Net" and "HumanEva-I" from the Discussion. We have replaced it with a new discussion focused on the specific performance of our proposed AGTM-Net and a detailed "Limitations" subsection (addressing Reviewer 2’s comments).

**Comments 5:** Scope of Parkinson’s disease symptoms should be addressed (limitations of Item 3.9)

**Response 5:** We agree that focusing on a single task is a limitation.

We have added a statement in the Discussion acknowledging that while MDS-UPDRS Item 3.9 is a critical indicator of axial impairment, a comprehensive PD diagnosis requires analyzing a broader range of motor tasks (e.g., gait, finger tapping).

**Comments 6:** Inconsistency between the “unified framework” claim and evaluation structure

**Response 6:** We have added the logical connection in the Introduction and Discussion.

We explain that the framework is conceptually unified: DATP acts as the necessary pre-processing module to reconstruct high-fidelity skeletons from imperfect clinical videos, which then serves as the high-quality input required by AGTM-Net for accurate disease classification.

**Comments 7:** Numerical inconsistencies between the Abstract and main text

**Response 7:** Thank you for spotting this error.

We have corrected the Abstract to match the experimental results reported in Table 1. The Abstract now correctly states that DATP achieves 77.72 PCK and 43.57 AUC (corrected from the previous erroneous values).

Response to Reviewer 2 Comments

**Point-by-point response to Comments and Suggestions for Authors**

**Comments 1:** You need to comment that a larger sample size would be of importance and that a multisite confirmation... would be warranted.

**Response 1**: We fully agree that the current sample size and single data source are limitations.

We have added a "Limitations" subsection in the Discussion. We explicitly state: "To establish broad clinical utility, larger sample sizes and multi-site validation are necessary to account for geographic variability and diverse patient demographics."

**Comments 2:** Greater variability of the stage of PD would be of great benefit... define disease trajectory.

**Response 2**: We fully agree that the current sample size and single data source are limitations.

We have added a "Limitations" subsection in the Discussion. We explicitly state: "To establish broad clinical utility, larger sample sizes and multi-site validation are necessary to account for geographic variability and diverse patient demographics."

**Comments 3:** You need to comment that this represents a preliminary analysis.

**Response 3**: We have explicitly included the sentence: "This study represents a preliminary analysis" at the beginning of the Discussion/Limitations section to properly frame the scope of our findings.
